# Development of a nanobody-based competitive enzyme-linked immunosorbent assay for the sensitive detection of antibodies against porcine deltacoronavirus

**DOI:** 10.1128/jcm.01615-24

**Published:** 2025-02-14

**Authors:** Ruiming Yu, Liping Zhang, Yingjie Bai, Peng Zhou, Jun Yang, Dongsheng Wang, Liyang Wei, Zhongwang Zhang, Chenghua Yan, Yonglu Wang, Huichen Guo, Li Pan, Ligang Yuan, Xinsheng Liu

**Affiliations:** 1 State Key Laboratory for Animal Disease Control and Prevention, Lanzhou Veterinary Research Institute, Chinese Academy of Agricultural Sciences 111658 , Lanzhou, China; 2 Gansu Province Research Center for Basic Disciplines of Pathogen Biology, Lanzhou, China; 3 College of Veterinary Medicine, Gansu Agricultural University 74661 , Lanzhou, China; 4 Hunan Institute of Animal and Veterinary Science, Changsha, China; 5 College of Traditional Chinese Medicine/College of Life Sciences, Jiangxi University of Traditional Chinese Medicine 74582 , Nanchang, China; University of California, Davis, Davis, California, USA

**Keywords:** porcine deltacoronavirus, nanobody, cELISA

## Abstract

Porcine deltacoronavirus (PDCoV) is an emerging porcine enteric coronavirus causing significant economic losses to the pig farming industry globally. In this study, we expressed the S protein of a highly virulent PDCoV strain in the CHO eukaryotic expression system. After immunizing alpaca with the PDCoV S protein and employing the phage display library technique, a high-affinity and specific nanobody Nb3 against PDCoV S protein was successfully established by three rounds of biopanning and a phage enzyme-linked immunosorbent assay (ELISA). Furthermore, a competitive ELISA (cELISA) was developed based on Nb3 to rapidly and efficiently detect PDCoV antibody levels. The cELISA displayed no cross-reaction with positive sera of porcine epidemic diarrhea virus (PEDV), transmissible gastroenteritis virus (TGEV), porcine rotavirus (PoRV), pseudorabies virus (PRV), classical swine fever virus (CSFV), porcine reproductive and respiratory syndrome virus (PRRSV), or porcine circovirus 2 (PCV2), thereby showing good specificity. The cELISA successfully detected positive sera diluted 1:127 (percentage inhibition ≥ 50.02%), indicating high sensitivity. Both the intra- and inter-batch coefficients of variation were less than 10%, indicating good repeatability. The cELISA had a total coincidence rate of 98.33% with the indirect immunofluorescence assay and a significant positive correlation with the virus neutralization test (*r* = 0.861, *P* < 0.001), suggesting that the cELISA can be used to measure the neutralizing antibody titers in serum samples. In conclusion, our nanobody-based cELISA showed good performance indicators and can be used to monitor and evaluate antibody levels following clinical infection of PDCoV or vaccine immunization.

**IMPORTANCE:**

This study screened out a high-affinity and specific nanobody Nb3 against porcine deltacoronavirus (PDCoV) S protein and established a nanobody-based competitive ELISA (cELISA) for PDCoV antibody detection. This cELISA is a simple, rapid, and specific method that can effectively measure the neutralizing antibody titers in serum samples.

## INTRODUCTION

Porcine deltacoronavirus (PDCoV), one of the predominant pathogens causing viral diarrhea in piglets (1–3), is a single-stranded RNA virus belonging to the family *Coronaviridae* of the genus *Deltacoronavirus*. The viral particles are approximately 100 nm in diameter and contain a genome of approximately 25.4 kb (4). The genome comprises a 5′ untranslated region (UTR), 3′UTR, and eight open reading frames encoding two polyproteins (pp1a and pp1b), four structural proteins [S (spike glycoprotein), E (envelope protein), M (membrane protein), and N (nucleoprotein protein)], and two non-structural proteins (NS6 and NS7) (5). PDCoV S protein is the key protein for invading host cells and plays an important role in virulence, tissue tropism, and the induction of neutralizing antibodies in the host; it is also the preferred target antigen for vaccine development (6–8).

PDCoV, which was first discovered in 2012 (9), has spread worldwide and seriously endangers the pig farming industry in many countries (4, 10). PDCoV can infect pigs of different ages and presents with similar clinical symptoms to porcine epidemic diarrhea virus (PEDV), including diarrhea, vomiting, and dehydration (1, 11–13). Previous studies have shown that the positive detection rate of PDCoV is second only to PEDV (14, 15). To date, a number of techniques have been applied for the detection and diagnosis of PDCoV, including virus isolation and identification, cytopathic effect observation, real-time fluorescence quantitative polymerase chain reaction (PCR), indirect fluorescent antibody assay, immunohistochemistry, and *in situ* hybridization (16). Enzyme-linked immunosorbent assay (ELISA) is considered the most sensitive and specific method for serum antibody detection (16). Therefore, establishing a rapid, sensitive, and specific method for PDCoV antibody detection is of great significance for the diagnosis, prevention, and control of PDCoV.

Animals belonging to the *Camelidae* family possess heavy chain-only antibodies (HCAbs) that naturally lack light chains and the CH1 domain of heavy chains. HCAbs consist of the CH2 and CH3 domains of heavy chains and the hinge and variable domains of the heavy chain of the heavy-chain antibody (VHH) (17). A single-domain antibody composed only of VHH can be obtained by cloning VHH with a crystal diameter of 2.5 nm, a length of 4 nm, and a relative molecular weight of only ~15 kDa, which is also known as a nanobody (Nb). A Nb is the smallest antigen-binding fragment currently known with complete antigen-binding capacity (18, 19). Nbs can bind antigens more efficiently than monoclonal antibodies (20). In addition, Nbs are characterized by a simple structure, easy modification, low immunogenicity, high stability, specificity, and affinity, and the potential for high expression (21). At present, Nbs are receiving widespread attention in the fields of human disease diagnosis, treatment, and research and are being clinically applied in the treatment of novel coronaviruses (22).

In this study, a library of specific Nbs against PDCoV S protein was constructed using the phage display library technique, and a high-affinity and specific Nb against PDCoV S protein was obtained. Furthermore, a competitive ELISA (cELISA) was developed based on Nb3 to rapidly and efficiently detect PDCoV antibody levels. This method offered low cost, high sensitivity, and strong specificity.

In summary, an Nb with a high binding activity was screened out and used to establish a cELISA for PDCoV antibodies, providing a new method for the clinical diagnosis of PDCoV.

## MATERIALS AND METHODS

### Materials

ExpiCHO-S cells, expression medium, and transfection reagent were purchased from Thermo Fisher Scientific. pcDNA3.1(+) expression vector, pComb3XTT, *Escherichia coli* TG 1, and helper phage M13KO7 were preserved by the State Key Laboratory of Animal Disease Control and Prevention, Lanzhou Veterinary Research Institute, China Academy of Agricultural Sciences. Alpaca IgG (H+L) horseradish peroxidase (HRP) was purchased from Jackson ImmunoResearch. The HRP labeling kit (BF06095S) was bought from Biodragon. The 300 pig serum samples were stored in our laboratory and included 120 PDCoV-infected piglet serum samples, 80 PDCoV-inactivated immune serum samples, and 100 non-PDCoV-infected serum samples positive for porcine epidemic diarrhea virus (PEDV), transmissible gastroenteritis virus (TGEV), porcine rotavirus (PoRV), pseudorabies virus (PRV), classical swine fever virus (CSFV), porcine reproductive and respiratory syndrome virus (PRRSV), and porcine circovirus 2 (PCV2) from unimmunized pigs. Alpacas were purchased from a farm.

### Methods

#### Expression and purification of PDCoV S protein

Following transfection with pCDNA3.1(+)–PDCoV-S recombinant plasmid, ExpiCHO-S cells were incubated at 32°C in a 5% CO_2_ incubator with humidified air for 10–12 days. Next, the medium was centrifuged at 4,000–5,000 × *g* for 30 min using a refrigerated centrifuge, and the supernatant was filtered through a 0.22 µm filter. The resulting supernatant was mixed with Ni-NTA agarose (Qiagen, Hilden, Germany) at 4°C for 2 h of binding, then the non-specifically bound impure protein was eluted with 1× phosphate-buffered saline (PBS), and the target protein was eluted with 1× PBS containing 500 mM imidazole. Thereafter, the protein samples with high purity were collected, and the protein concentration was measured using a BCA Protein Assay Kit. The protein samples were then diluted to 1 mg/mL, filtered with a 0.22 µm filter, and aseptically dispensed, followed by short-term storage at 4°C and long-term storage at −80°C prior to subsequent use.

#### Immunization of alpacas with PDCoV S protein

Two alpacas aged 1–2 years were immunized following the standard immunization protocol for protein antigens. Specifically, blood samples were collected prior to immunization, and 2 mL of alpaca negative serum was prepared. In the first immunization, PDCoV S antigen (1 mg) was mixed with Freund’s complete adjuvant at a 1:1 ratio, emulsified, and then injected subcutaneously at multiple points. Booster immunization was carried out with a mixture of PDCoV S antigen (0.5 mg) with Freund’s incomplete adjuvant at a ratio of 1:1 at 21, 42, and 63 days after the first immunization. At 28, 49, and 70 days after the first immunization, the peripheral blood of alpacas was collected, and the serum was separated to measure the PDCoV S-specific antibody potency.

#### Construction of the Nb library

**Fig 1:**
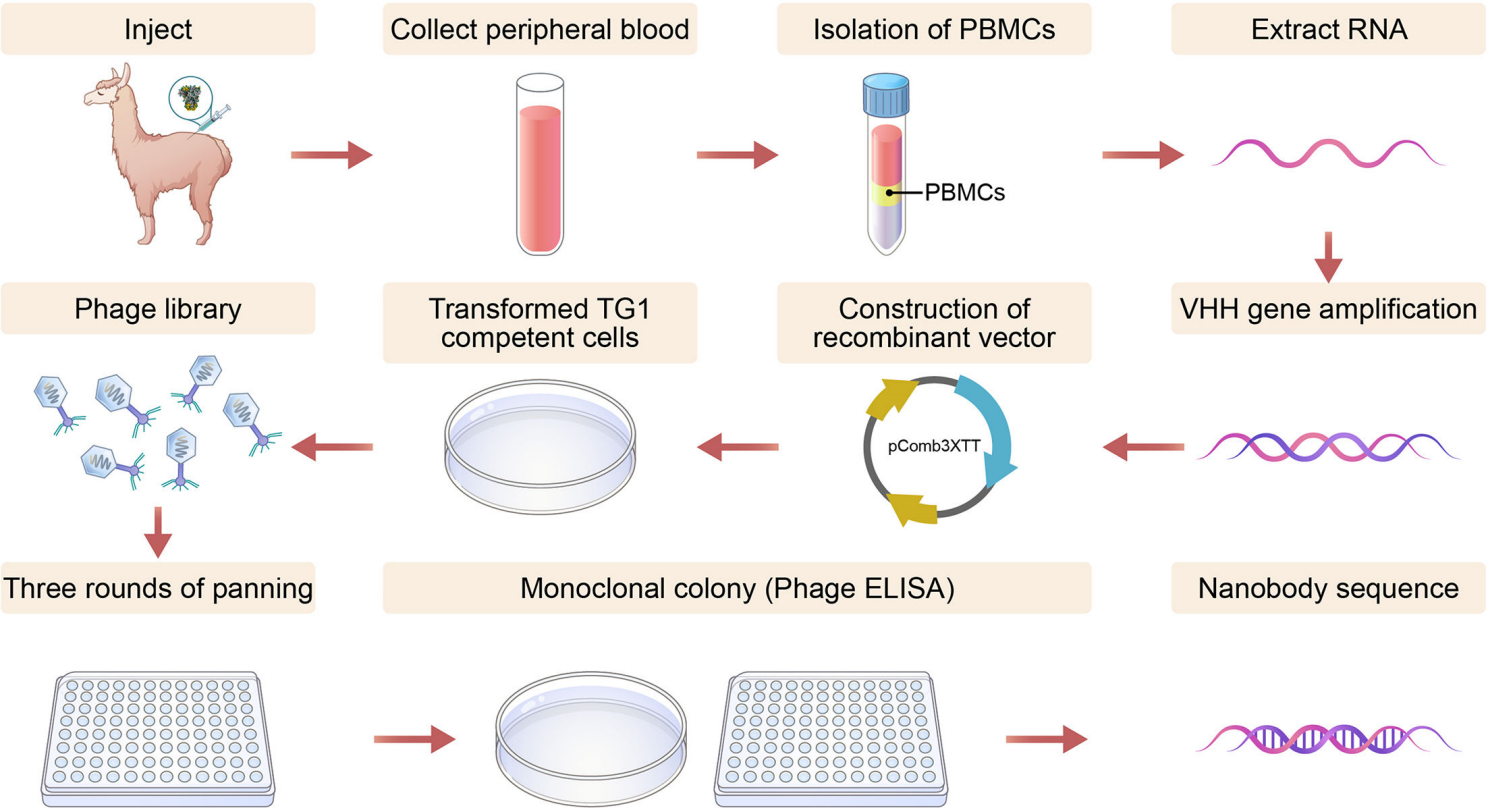
Pattern diagram of nanobody preparation using phage display technology. First, the alpaca was immunized, PBMCs were isolated, the *VHH* gene was amplified, and the nanobody library was constructed using phage display technology. Then, specific nanobodies were screened by biopanning, phage ELISA, and soluble ELISA.

At 70 days after the first immunization, peripheral blood was collected from alpacas, from which peripheral blood mononuclear cells (PBMCs) were isolated, and RNA was extracted and prepared into cDNA for construction of the Nb library (23, 24) (Fig. 1). In detail, the cDNA was used as a template to amplify the heavy chain antibody gene of alpaca with the heavy chain antibody primer set (HcAb-F/HcAb-R). The PCR product was loaded onto an agarose gel and separated by electrophoresis. The target fragment (500–750 bp) was excised and purified using a gel recovery kit. Then, the VHH gene library fragment was amplified using VHH-specific primers (VHH-F/VHH-R) and cloned into the pComb3XTT vector, which was then electrotransformed into *E. coli* TG1-competent cells. Then, a series of 10-fold dilutions was generated from 10 µL of the transformation mixture, and ampicillin (Amp)-resistant plate counts were performed on three gradients of 10^−4^, 10^˗5^, and 10^˗6^ to assess the library capacity: capacity = number of clones × dilution factor × total vol of transformed product (mL). Then, 30 monoclones were randomly selected for sequencing and assessed for the insertion rate and diversity. The positive clones were plated on Amp-resistant plates and cultured overnight, scraped, and mixed the next day. Finally, glycerol was added at a final concentration of 20%, and aliquots were stored at −80°C prior to use.

### Biopanning of PDCoV S protein-specific recombinant phage

Specific recombinant phage was screened using PDCoV S protein as a coating antigen. Specifically, PDCoV S protein was diluted to 100 µg/mL with carbonate buffer, added to a 96-well ELISA plate at 100 µL/well, and coated overnight at 4°C. The next day, the coating solution in wells was discarded, and 200 µL of blocking buffer was added to each well for blocking at 37°C for 2 h. After the solution in the wells was discarded, the plate was washed five times with PBS containing Tween-20 (PBST), and then 100 µL of diluted phage library was added to each well, followed by incubation at 37°C for 1 h. After discarding the supernatant, the plate was washed 10 times with PBST, and then 100 µL of 0.1 M triethylamine solution was added to each well, followed by incubation at room temperature for 10 min. The eluent in wells was aspirated and neutralized with an equal volume of 1 M Tris–HCl (pH 7.4). Then, the phage titer was measured, and the yield was calculated, followed by amplification culture and the next round of biopanning. A total of three rounds of biopanning were conducted.

### Phage ELISA

PDCoV S protein was used to coat a 96-well ELISA plate (10 ng/well) overnight at 4°C. The wells were then blocked with 2% skim milk powder at 37°C for 1 h, and 100 µL of phage supernatant was added to each well, followed by incubation at 37°C for 1 h. After washing three times with PBST, 100 µL of HRP-labeled anti-M13 antibody (1:15,000) was added to each well and incubated at 37°C for 1 h. After washing three times with PBST, 100 µL of TMB chromogenic solution was added to each well for development at 37°C for 15 min. Thereafter, 100 µL of stop buffer (2 M H_2_SO_4_) was added to each well, and the optical density at 450 nm (OD_450 nm_) was measured by a microplate reader. Positive clones obtained by phage ELISA screening were selected and retested by phage ELISA with PDCoV S protein, PDCoV, and multi-tag protein (containing HA and His tags) for validation.

### Soluble ELISA

PDCoV S protein, PDCoV, or His-tag protein were used to coat a 96-well ELISA plate overnight at 4°C. The wells were then blocked with 2% skim milk powder at 37°C for 1 h and 100 µL of supernatant was added to the periplasmic space in each well, and incubated at 37°C for 1 h. The plate was washed three times with PBST, and 100 µL of HRP-labeled anti-HA tag antibody (1:4000) was added to each well and incubated at 37°C for 1 h. The plate was washed three times with PBST, and 100 µL of TMB chromogenic solution was added to each well for development at 37°C for 15 min. Finally, 100 µL of stop buffer (2 M H_2_SO_4_) was added to each well, and the OD_450 nm_ was measured using a microplate reader.

### Expression and purification of the Nb

The resulting VHH gene fragments were cloned into the pCold II expression vector and transformed into *E. coli* BL21 (DE3) competent cells. Monoclones were inoculated into 5–10 mL of lysogeny broth (LB) liquid medium containing 100 µg/mL Amp and incubated overnight at 37°C with shaking at 220 rpm. The overnight culture was then inoculated into 500 mL of LB medium at a 1:100 ratio for the induction of expression at low temperature (15°C). After 24 h, the phage precipitate collected was resuspended in PBS (20 mL PBS/g wet weight of phage) and subjected to ultrasonication (300 W for 3 s, 3 s, and 40 min in an ice water bath). Then, centrifugation was performed at 10, 000 × *g* for 30 min, and the purified protein in the supernatant was finally collected and labeled with HRP using a HRP Labeling Kit (BF06095S, Biodragon).

### Specificity analysis and titer determination of the Nb

The specificity and titer of Nb were detected by indirect ELISA to test cross-reactivity with five different viral proteins (PDCoV-S, PDCoV-N, PEDV-S, PEDV-N, and His-tag proteins). Briefly, equal amounts of the five proteins were used to coat 96-well plates and were then incubated overnight at 4°C. After blocking with 5% bovine serum albumin (BSA), the HRP-labeled Nb was diluted to different concentrations (4,000, 400, 40, 20, 10, 5, 2.5, and 1.25 ng/ml) and incubated with antigen at 37°C for 1 h, followed by washing five times with PBST. Afterwards, 100 µL of TMB solution was added to each well and incubated at 37°C for 15 min. Then, the reaction was stopped by adding 100 µL of 2 M H_2_SO_4_ to each well, and the OD_450 nm_ was finally measured using a microplate reader.

### Development of cELISA

A cELISA for PDCoV antibodies was established using HRP-labeled Nb. The optimal conditions for the ELISA were screened by the square titration method for the optimization of the antigen coating concentration (10, 5, 2.5, 2.0, 1.0, 0.5, and 0.2 µg/mL), antigen coating conditions (37°C for 1 h, 37°C for 2 h, and 4°C for 12 h), blocking conditions (37°C for 1 h, 37°C for 2 h, and 4°C for 12 h), serum dilution ratio (1:1, 1:2, 1:3, 1:5, 1:9, 1:19, and 1:29), Nb3-HRP dilution (1:2,500, 1:5,000, 1:10,000, 1:15,000, 1:20,000, and 1:40,000), competitive reaction time (30, 60, 90, and 120 min at 37°C), and TMB development time (5, 10, and 15 min at 37°C). The reaction conditions with a higher percentage inhibition (PI) and reasonable negative and positive values were selected as the optimal reaction conditions for the cELISA [PI = (1 − positive serum OD_450 nm_/negative serum OD_450 nm_) × 100%].

### Determination of the cutoff values

In total, 94 PDCoV-negative sera confirmed by the indirect immunofluorescence assay (IFA) were tested twice by the optimized cELISA, and the PI was calculated for each sample. The mean value (*X*) and standard deviation (SD) of the PI of the 94 samples were calculated. The two threshold cutoff values were defined as the mean PI value of the negative samples + 2 or 3 × SDs. Therefore, PI ≥ *X* + 3 SD indicated a positive result; PI ≤ *X* + 2 SD indicated a negative result; and *X* + 2 SD < PI < *X* + 3 SD was considered inconclusive.

### Determination of the specificity, sensitivity, and repeatability of the cELISA

The established cELISA was used to detect the sera positive for PEDV, TGEV, PoRV, PRV, CSFV, PRRSV, and PCV2 stored in our laboratory, with three replicates for each sample, and a PDCoV-positive control was included to verify the specificity of the cELISA. Moreover, three PDCoV-positive sera diluted at 1:3, 1:7, 1:15, 1:31, 1:63, 1:127, and 1:255 were subjected to the cELISA, with three replicates for each sample, and a negative control was included to verify the sensitivity of the cELISA. To test intra-batch repeatability, purified PDCoV S protein was coated with carbonate coating buffer on one ELISA plate, and six pig sera with clear backgrounds were randomly selected for detection, with three replicates for each sample; six repeatability tests were conducted in total. To test inter-batch repeatability, samples from different time points were used to coat three ELISA plates, and cELISA was performed on six pig sera, with three replicates for each sample.

### Immunofluorescence assay

Because there is no commercially available kit for PDCoV antibody detection, the pig sera were tested for PDCoV antibody by an IFA in this study. Specifically, LLC-PK1 cells were infected with PDCoV (multiplicity of infection = 0.01) in a 96-well plate upon reaching 80% confluence. After 24 h, the medium was discarded, and the sample was washed three times with PBS, fixed with 4% paraformaldehyde at 4°C for 1 h, permeabilized with 0.25% Triton-100 at room temperature for 10 min, and blocked with 5% BSA for 1 h. Then, the sample was incubated with pig serum and 488-labeled goat anti-porcine IgG as primary and secondary antibodies, respectively, stained with 4ʹ,6-diamino-2-phenylindole for 5 min away from light, and washed three times with PBS, followed by observation under a fluorescence microscope.

### Virus neutralization test (VNT)

The PDCoV neutralizing antibody titer in sera was measured using the virus fixation–serum dilution method. Specifically, the serum was inactivated in a water bath at 56°C for 30 min and serially diluted 2-fold in a 96-well plate, with four replicates for each dilution. An equal volume of diluted virus solution [200 50% tissue culture infective dose (TCID_50_)] was added into each well, mixed well, and left to stand in a 5% CO_2_ incubator at 37°C for 1 h. A virus control at 200 TCID_50_ (all lesions), 20 TCID_50_, 2 TCID_50_, and 0.2 TCID_50_ (no lesions) was included, along with a normal control. After incubation, 100 µL of virus-serum mixture was added into each well of a 96-well plate upon reaching 70–80% of confluence, and the plate was incubated in a 5% CO_2_ incubator at 37°C for 1 h. The solution in the wells was discarded, and the plate was washed three times with PBS, then 200 µL of maintenance medium (containing 20 µg/mL trypsin) was added to each well. Over 5–7 days of observation, the cytopathic effect was recorded.

## RESULTS

### Expression, purification, and identification of PDCoV S protein

Ni column affinity chromatography was used to purify the recombinant protein. The resulting protein was subjected to sodium dodecyl sulfate–polyacrylamide gel electrophoresis (SDS-PAGE). Following Coomassie brilliant blue staining, a clear target protein band was observed between 150 and 250 kDa. The protein size matched the expected range, and the purity was high (Fig. S1A). In addition, the recombinant protein was examined by western blotting with mouse anti-PDCoV S monoclonal antibody, and this antibody specifically recognized the purified PDCoV S recombinant protein (Fig. S1B).

### Construction of the VHH phage display library

After five immunizations, the potency of alpaca IgG(H + L) against PDCoV S in alpaca sera detected by indirect ELISA was 1:9,841,500 (Fig. S1C), which was eligible for subsequent library construction. The alpaca antibody fragment obtained by PCR amplification showed a target band of approximately 500–750 bp (Fig. S2A). Furthermore, the VHH library fragments were amplified, and a target band with an expected size (~400 bp) was yielded by PCR amplification (Fig. S2B). The Amp-resistant plate counts revealed that the mean capacity was (300 × 10 × 10^4 + 2^ + 29 × 10 × 10^5 + 2^) = 2.95 × 10^9^ (Fig. S2C). Thirty monoclones were randomly selected for sequencing, resulting in 29 valid clones (No. 1 clone showed no electrophoretic band) (Fig. S2D). Amino acid sequence alignment revealed that 28 out of the 29 clones contained antibody fragments in the correct reading frame (Fig. S2E). The total accuracy of the library was ~28 ÷ 30 × 100% = 93.3%. A neighbor-joining tree of VHH fragments was constructed using MEGA software, and the results revealed a diversity of VHH fragments (Fig. S2F), suggesting a high-quality library.

### Screening and identification of the PDCoV S protein-specific Nb library

Specific Nb was screened using purified PDCoV S protein as a coating antigen. During the screening process, the enrichment effect of specific VHH recombinant phage was assessed by detecting the recombinant phage titer in each round of eluate. The results showed that the phage recovery rate increased throughout the three rounds, and the P/N value reached 7,500 after the third round, suggesting a significant enrichment of specific recombinant phage (Table 1).

**TABLE 1:** Enrichment of nanobodies against PDCoV-S protein specific phages during three rounds of panning^*a*^

Round of panning	Input phage (PFU/well)	P output (PFU/well)	N output (PFU/well)	Recovery (P/input)	P/N
1st round	1.2 × 10^12^	1.8 × 10^7^	7.2 × 10^6^	1.5 × 10^−5^	2.5
2nd round	1.2 × 10^12^	4.5 × 10^9^	5.4 × 10^6^	3.75 × 10^−3^	8.33 × 10^2^
3rd round	1.2 × 10^12^	2.4 × 10^10^	3.2 × 10^6^	2.0 × 10^−2^	7.5 × 10^3^

^*a*^ The number of phages added before each round of screening is recorded as input; the number of phages harvested from positive enzyme-labeled holes after screening is recorded as P; and the number of phages harvested from negative control holes is recorded as N. The P/input represents the recovery rate. The higher the P/N value, the higher the proportion of specific phages.

After the third round of screening, 940 monoclones were randomly selected for phage ELISA, from which 153 positive clones were screened for retesting (Fig. S3A). The positive clones, together with virus and multi-tag protein (containing HA and His tags), were retested by phage ELISA. Finally, 22 positive clones were obtained (Fig. S3B). Fourteen unique sequences were obtained following PCR amplification and sequencing. After soluble ELISA, three positive clones (Nb1, Nb3, and Nb8) binding to both PDCoV S protein and virus were obtained (Fig. S3C).

### Analysis of Nb3 expression, purification, and specificity

**Fig 2:**
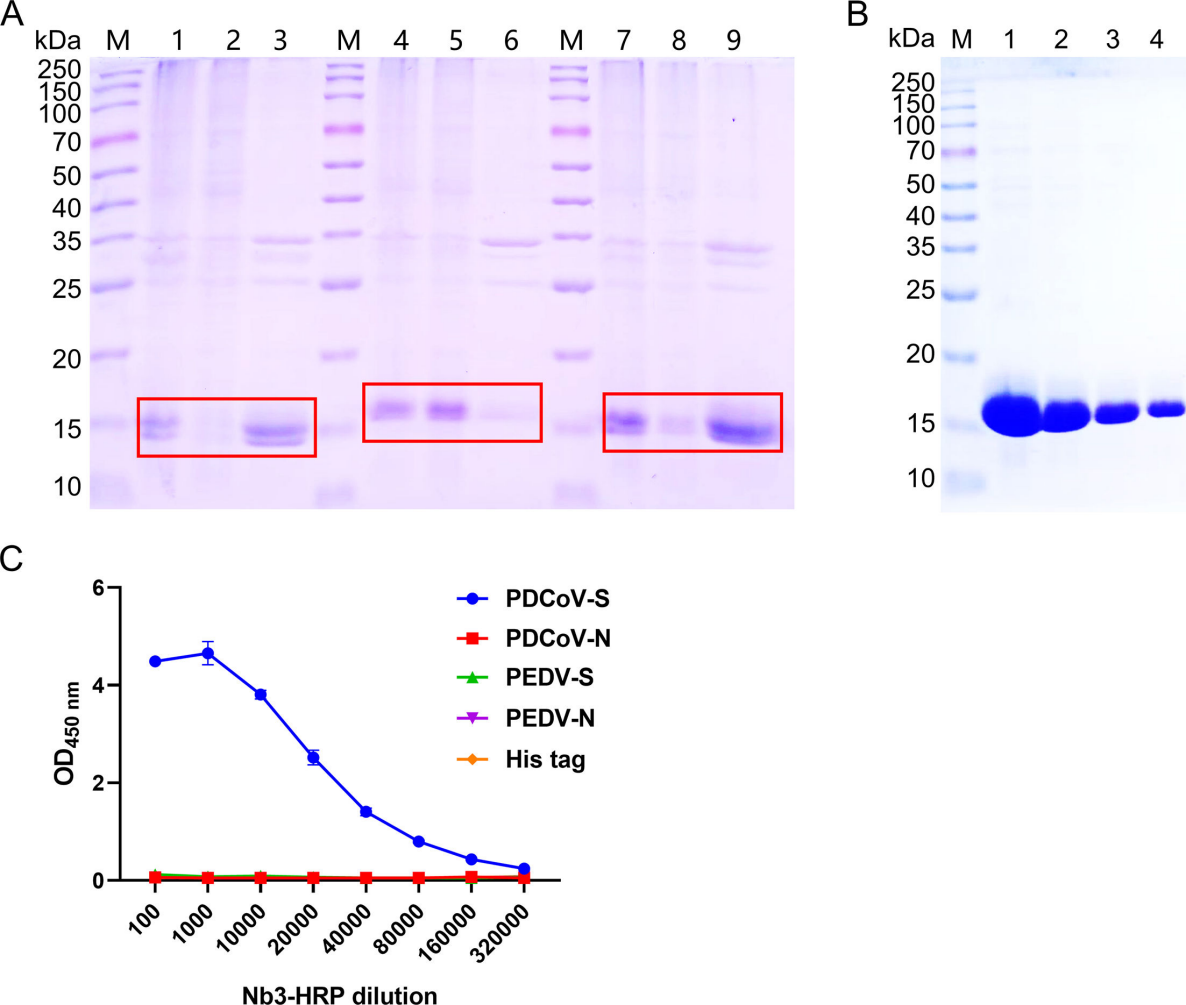
Expression, purification, and specificity analysis of nanobodies. (**A**) Solubility analysis of Nb1 (lane 1: lysates of Nb1 whole bacteria; lane 2: ultrasonic supernatant of Nb1 bacteria; lane 3: inclusion body lysate of Nb1 bacteria), Nb3 (lane 4: lysates of Nb3 whole bacteria; lane 5: ultrasonic supernatant of Nb3 bacteria; lane 6: inclusion body lysate of Nb3 bacteria), and Nb8 (lane 7: lysates of Nb8 whole bacteria; lane 8: ultrasonic supernatant of Nb8 bacteria; lane 9: inclusion body lysate of Nb8 bacteria). (**B**) The purified Nb3 was detected by SDS-PAGE. (**C**) The specificity and titer of Nb3-HRP were determined by indirect ELISA.

SDS-PAGE revealed that the expressed and purified Nbs (Nb1, Nb3, and Nb8) were consistent with the expected molecular weights (Fig. 2A). Nb3 was expressed in soluble form in the supernatant, whereas Nb1 and Nb8 were expressed in insoluble inclusion body form. Therefore, Nb3 was selected for further purification by Ni column affinity chromatography. The results showed that Nb3 had high expression and purity (Fig. 2B). Nb3 was labeled with HRP using the HRP Labeling Kit (BF06095S, Biodragon). The specificity and potency of Nb3-HRP were determined by indirect ELISA. It was found that Nb3 only reacted with PDCoV S protein, indicating good specificity with potency up to 1:160,000 (Fig. 2C).

### Optimal conditions of cELISA for PDCoV antibody detection

Using the square titration method, the reaction conditions with a higher PI and reasonable negative and positive values were selected as the optimal reaction conditions for cELISA. The selected conditions were as follows: optimal coating concentration: 200 ng/well at 37°C for 2 h, optimal blocking condition: 37°C for 2 h, optimal serum dilution ratio: 1:3, optimal Nb3-HRP dilution: 1:10,000, optimal competitive reaction time: 120 min at 37°C, and optimal TMB development condition: 37°C for 10 min away from light.

### Determination of cutoff values

Ninety-four PDCoV-negative sera confirmed by IFA were tested, and the PI was calculated. PI ≥ 50.02% indicated a positive result; PI ≤ 40.29% indicated a negative result; and 40.29% < PI < 50.02% was considered inconclusive.

### Specificity, sensitivity, and repeatability of cELISA

**Fig 3:**
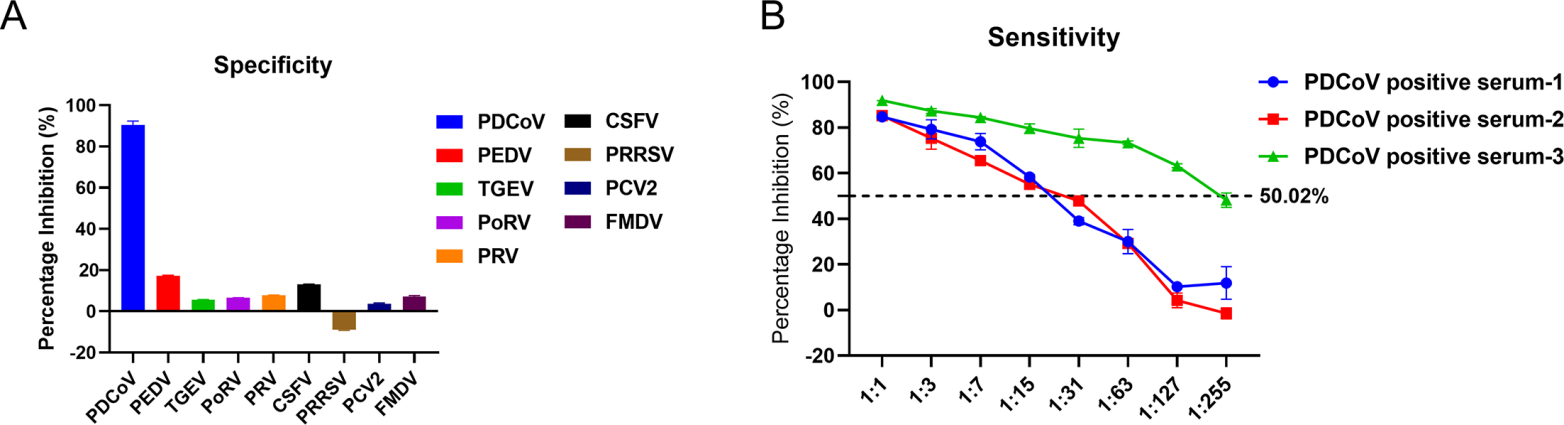
Specificity and sensitivity analysis of the cELISA. (**A**) The positive sera of PEDV, TGEV, PoRV, PRV, CSFV, PRRSV, and PCV2 were detected by cELISA, and the PI values were less than 40.29%, indicating that the method had good specificity. (**B**) Three positive sera continued to test positive when diluted at 1:15, 1:15, and 1:127 (PI ≥ 50.02%), indicating that the method had high sensitivity.

The results of the specificity test of positive sera of common swine diseases showed that the positive sera of PEDV, TGEV, PoRV, PRV, CSFV, PRRSV, and PCV2 had a PI < 40.29% (Fig. 3A), indicating the good specificity of the cELISA. Moreover, three positive sera continued to test positive (PI ≥ 50.02%) when diluted at 1:15, 1:15, and 1:127 (Fig. 3B), indicating the high sensitivity of the cELISA. In addition, both intra- and inter-batch coefficients of variation were less than 10% (Table 2), indicating good repeatability of the cELISA.

**TABLE 2:** Reproducibility of the cELISA determined by intra- and inter-assay CV values

Sample no.	Intra-assay			Inter-assay	
	PI mean ± SD	CV/%		PI mean ± SD	CV/%
1	86.08 ± 0.37	0.42		77.43 ± 1.08	1.39
2	69.50 ± 0.53	0.76		62.98 ± 1.15	1.82
3	83.73 ± 0.87	1.04		84.00 ± 0.74	0.88
4	19.62 ± 0.61	3.10		23.98 ± 0.71	2.98
5	17.88 ± 0.42	2.33		17.48 ± 0.44	2.49
6	3.87 ± 0.11	2.79		7.32 ± 0.31	4.25

### Determination of PDCoV antibody-positive and -negative sera

**Fig 4:**
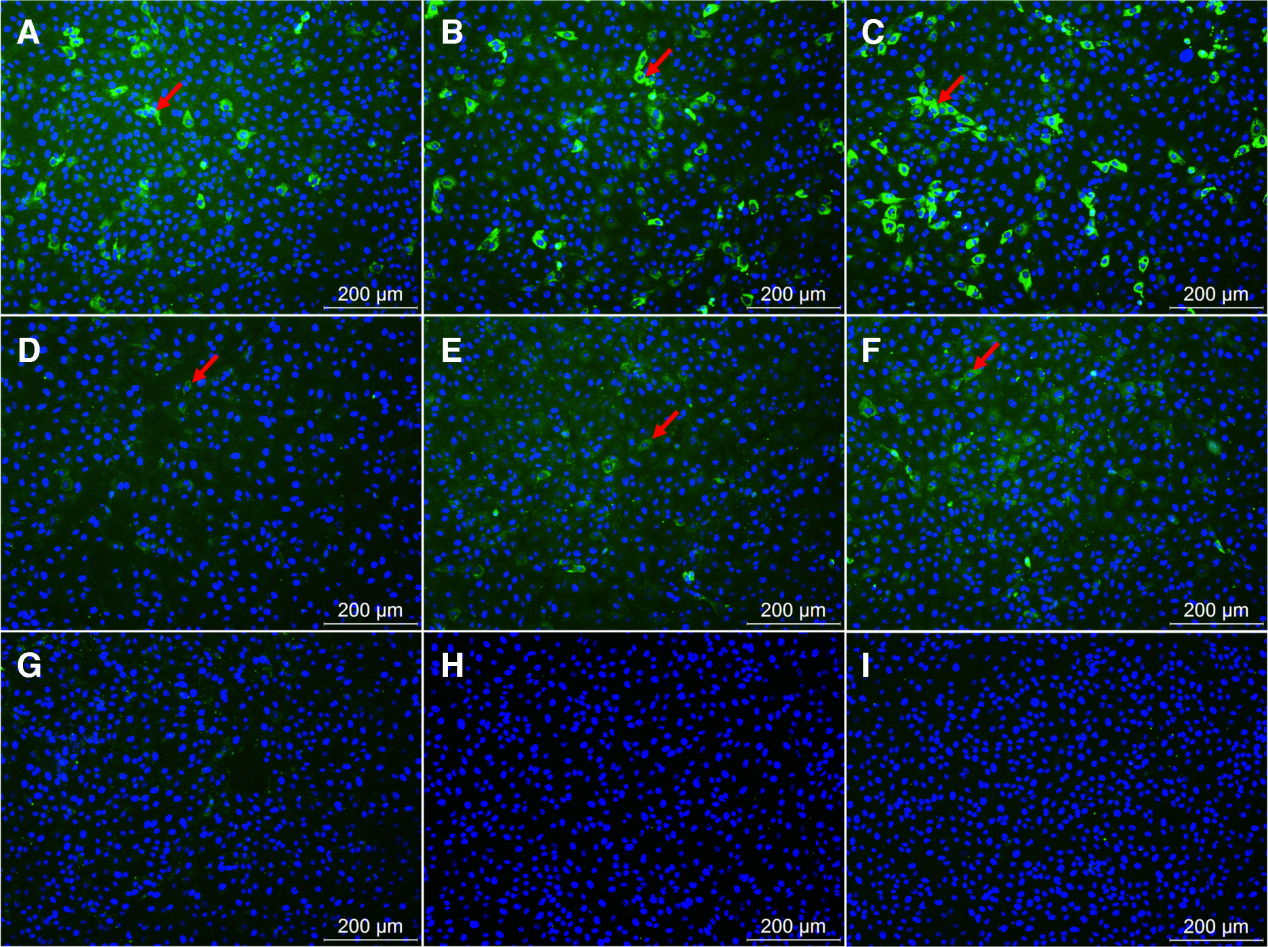
Identification of PDCoV-negative and -positive sera by an immunofluorescence assay. (A–C) Strongly positive serum samples showed high-intensity fluorescence. (D–F) Weakly positive serum samples showed low-intensity fluorescence. (G–I) Negative serum samples showed no visible fluorescence in the immunofluorescence assay.

IFA was used to detect 300 pig sera preserved in our laboratory. The results indicated 94 negative sera (Fig. 4G through I) and 206 positive sera, including strongly positive sera (Fig. 4A through C) and weakly positive sera (Fig. 4D through F). The results of the cELISA revealed 95 negative sera and 205 positive sera. Therefore, the cELISA had a total coincidence rate of 98.33% with IFA (Table 3).

**TABLE 3:** Detection of porcine serum and comparison results with IFA

		cELISA		Total	Agreement rate/%
		Positive	Negative		
IFA	Positive	203	3	206	98.54
	Negative	2	92	94	97.87
Total		205	95	300	98.33

### Significant correlation between the cELISA and the VNT

**Fig 5:**
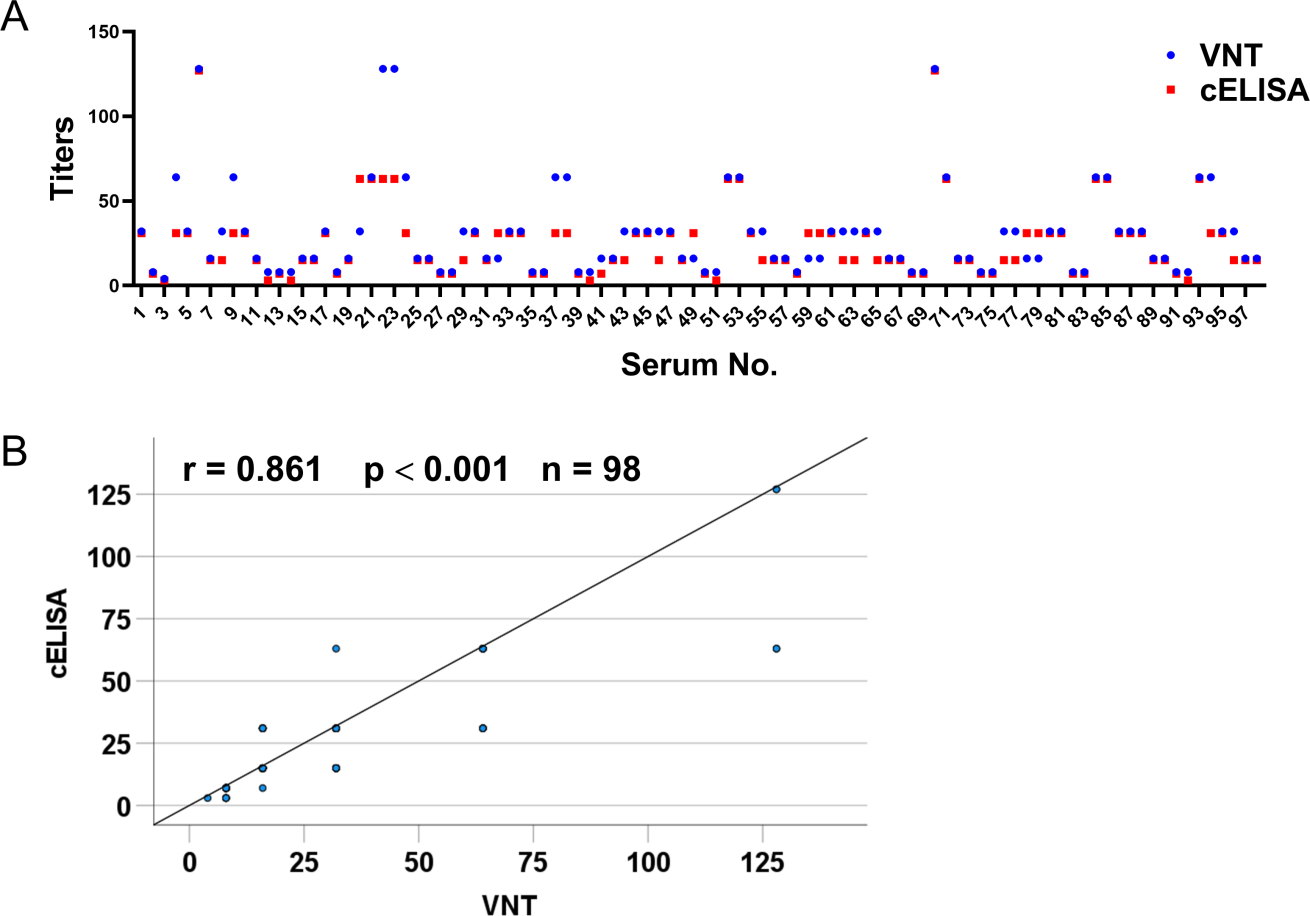
Correlation analysis between the cELISA and VNT titers. (**A**) Dispersion of individual values obtained using cELISA and VNT. (**B**) Correlation between the cELISA and VNT titers. The correlation between cELISA and VNT titers was tested by Pearson’s coefficient using IBM SPSS software, and *P* < 0.05 was considered statistically significant (*r*: correlation coefficient, *n*: number of serum samples tested). The *P* value was two-tailed. Note: some dots represent more than one serum sample.

In total, 98 PDCoV-infected pig sera were tested for the antibody endpoint titer by the cELISA and VNT. As shown in Fig. 5A, the neutralizing antibody titer of sera displayed a significant positive correlation with the antibody titer measured by the cELISA (*i.e*., the higher the neutralizing antibody titer, the higher the antibody titer measured by the cELISA). Moreover, an extremely significant positive correlation was found between the cELISA and VNT by calculating the Pearson’s correlation coefficient (*r* = 0.861, *P* < 0.001) (Fig. 5B).

## DISCUSSION

PDCoV displays a lethality rate of over 80% in suckling piglets, thereby causing considerable economic damage to the pig farming industry. As a result of cross-infection and highly similar clinical symptoms to PEDV, TGEV, and other porcine enteroviruses (25), PDCoV is mainly diagnosed through virological and serological methods (4). As a serological method, ELISA has been an important and effective tool in epidemiological investigations, being able to provide information about the history of PDCoV exposure and also determine antibody responses to infections or vaccinations. Currently, many types of ELISA are being developed for PDCoV antibodies, such as indirect ELISA based on PDCoV N, M, or S protein (26–30). However, indirect ELISA is prone to serum cross-reaction (31, 32) and requires enzyme-labeled secondary antibodies of corresponding genera, which is inconvenient for detecting sera of different genera. By contrast, cELISA can effectively avoid these drawbacks.

Monoclonal antibodies are mainly used by traditional ELISA kits, but they have shortcomings, such as long production times, difficulty in purification and labeling of antibodies, and high costs, which hinder the development of commercial ELISA kits (33). By comparison, Nbs do not have these limitations, so they are widely used in the development of ELISA methods (34). In recent years, considerable progress has been made in the research and development of Nbs as diagnostic products for infectious diseases. cELISA for PEDV (23), FMDV (35), ASFV (36, 37), and PCV2 (38) has been developed based on Nbs, showing higher specificity and sensitivity than commercially available kits. In particular, diagnostic sensitivity can be improved by Nb engineering. Ma et al. (23) developed a new blocking ELISA for the detection of antibodies using PEDV N protein-specific Nb, with sensitivity and specificity of 100 and 93.18%, respectively. This new method showed a coincidence rate of 94% with commercial ELISA kits based on 150 serum samples. In this study, the established cELISA also had high specificity, sensitivity, and repeatability, indicating the strong practicability of the Nb-based antibody detection method.

To prepare high-quality immune antibodies with strong specificity, high titer, and good affinity, it is necessary to select an appropriate antigen. Studies have shown that inactivated coronavirus particles are spherical or polyhedral in shape, with the distribution of spike proteins being uneven between different particles. There are two types of spike proteins on the surface: the larger complete S protein trimer and the smaller S2 trimer after membrane fusion; the fusion S protein is the predominant type (39). The existence of two types of proteins is not conducive to the induction of S protein-specific antibodies, especially neutralizing antibody levels. The S protein expressed by the ExpiCHO eukaryotic expression system has a large molecular weight, high purity, and strong immunogenicity, which can effectively induce alpaca to produce S protein-specific antibodies and neutralizing antibodies. This is conducive to the establishment of a high-quality Nb library.

Currently, the development of PDCoV-specific antibody detection methods remains in the research and development stage, and there are few commercially available detection kits. In this study, classical assay methods (IFA or VNT) were used to calibrate the test samples, which are also the technical methodologies selected by many researchers (30, 40). In this study, 300 pig sera were detected by IFA. The cELISA had a total coincidence rate of 98.33% with IFA. VNT is usually used for the detection of neutralizing antibodies, which is a complex and time-consuming process. A significant positive correlation was observed between the cELISA and VNT in this study (*r* = 0.861, *P* < 0.001), and the titer of neutralizing antibody was highly consistent with that measured by the cELISA. Therefore, the cELISA can be used to detect the neutralizing antibody titer in serum samples, reducing both time and costs.

In this study, alpacas were immunized with eukaryotic PDCoV S protein for the first time; a specific Nb library against PDCoV S protein was constructed using the phage display library technique; and PDCoV S protein-specific Nb was screened. Nb3 was expressed in a soluble form in the prokaryotic expression system. After eukaryotic expression and purification, Nb3 could bind to PDCoV S protein with high affinity and specificity. Based on Nb3, a cELISA for PDCoV antibody detection was established for the first time. The cELISA displayed no cross-reactivity with positive sera of PEDV, TGEV, PoRV, PRV, CSFV, PRRSV, and PCV2, confirming its high specificity. Moreover, three positive sera continued to test positive (PI ≥ 50.02%) when diluted at 1:15, 1:15, and 1:127, indicating high sensitivity. In addition, both intra- and inter-batch coefficients of variation were less than 10%, indicating good repeatability. In conclusion, cELISA is simple to operate and offers high sensitivity, specificity, and reproducibility, making it a promising technique for the clinical detection of PDCoV antibody levels in the future.

### Data analysis

All data were plotted using GraphPad Prism software. The correlation between cELISA titer and VNT titer was tested by Pearson’s coefficient using IBM SPSS software, and *P* < 0.05 was considered statistically significant.

## Data Availability

The data supporting the results of this study are available from the corresponding author upon reasonable request.

## SUPPLEMENTAL MATERIAL

Supplemental material jcm.01615-24-s0001.docx Table S1; Figures S1 to S3.

ASM does not own the copyrights to Supplemental Material that may be linked to, or accessed through, an article. The authors have granted ASM a non-exclusive, world-wide license to publish the Supplemental Material files. Please contact the corresponding author directly for reuse.
