# Supplementary material for: Development of a nanobody-based competitive enzyme-linked immunosorbent assay for the sensitive detection of antibodies against porcine deltacoronavirus
Source: J Clin Microbiol. 2025 Feb 14;63(3):e01615-24. doi: 10.1128/jcm.01615-24 (PMC11898664; doi:10.1128/jcm.01615-24)
Supplement: Supplemental material — Table S1; Figures S1 to S3. [file jcm.01615-24-s0001.docx]

**Development of a Nanobody-based Competitive Enzyme-linked Immunosorbent Assay for the Sensitive Detection of Antibodies against Porcine Deltacoronavirus**

Ruiming Yu^1,2,3^, Liping Zhang^1,2^, Yingjie Bai^1,2^, Peng Zhou^1,2^, Jun Yang^4^, Dongsheng Wang^1,2,3^, Liyang Wei^1,2^, Zhongwang Zhang^1,2^, Chenghua Yan^5^, Yonglu Wang^1,2^, Huichen Guo^1,2^, Pan Li^1,2^, Ligang Yuan^3^*, Xinsheng Liu^1,2^*

^1^ State Key Laboratory for Animal Disease Control and Prevention, Lanzhou Veterinary Research Institute, Chinese Academy of Agricultural Sciences, Lanzhou 730046, China.

^2^ Gansu Province Research Center for Basic Disciplines of Pathogen Biology, Lanzhou 730046, China.

^3^ College of Veterinary Medicine, Gansu Agricultural University, Lanzhou 730070, China.

^4^ Hunan Institute of Animal and Veterinary Science, Changsha, 410131, China.

^5^ College of Traditional Chinese Medicine/College of Life Sciences, Jiangxi University of Chinese Medicine, Nanchang, 330004, China.

Authors' email addresses:

Ruiming Yu: yuruiming1992@163.com

Liping Zhang: [zhangliping03@caas.cn](mailto:zhangliping03@caas.cn)

Yingjie Bai: [baiyingjay@163.com](mailto:baiyingjay@163.com)

Peng Zhou: [zhoupeng02@caas.cn](mailto:zhoupeng02@caas.cn)

Jun Yang: yangjunpro@163.com

Dongsheng Wang: [wangdongsheng0728@126.com](mailto:wangdongsheng0728@126.com)

Liyang Wei: 32134555211@qq.com

Zhongwang Zhang: [zhangzhongwang@caas.cn](mailto:zhangzhongwang@caas.cn)

Chenghua Yan: yanchenghua23@126.com

Yonglu Wang: wangyonglumd@hotmail.com

Huichen Guo: guohuichen@caas.cn

Li Pan: [panli@caas.cn](mailto:panli@caas.cn)

Ligang Yuan* (corresponding author): yuan2918@126.com

Xinsheng Liu* (corresponding author): liuxinsheng@caas.cn

**Table S1 Primer pairs in the study.**

| **Names** | **Sequences (5' → 3')** |
| --- | --- |
| HcAb-F | GGTGGTCCTGGCTGCTCTTTTACA |
| HcAb-R | GAAGAGTGTCACGCTGGGGGGCAG |
| VHH-F | CAGGTGCAGCTGGCGGAGTCGGG |
| VHH-R | TTGTGGTTTTGGTGTCTTGGGTTCTGAG |


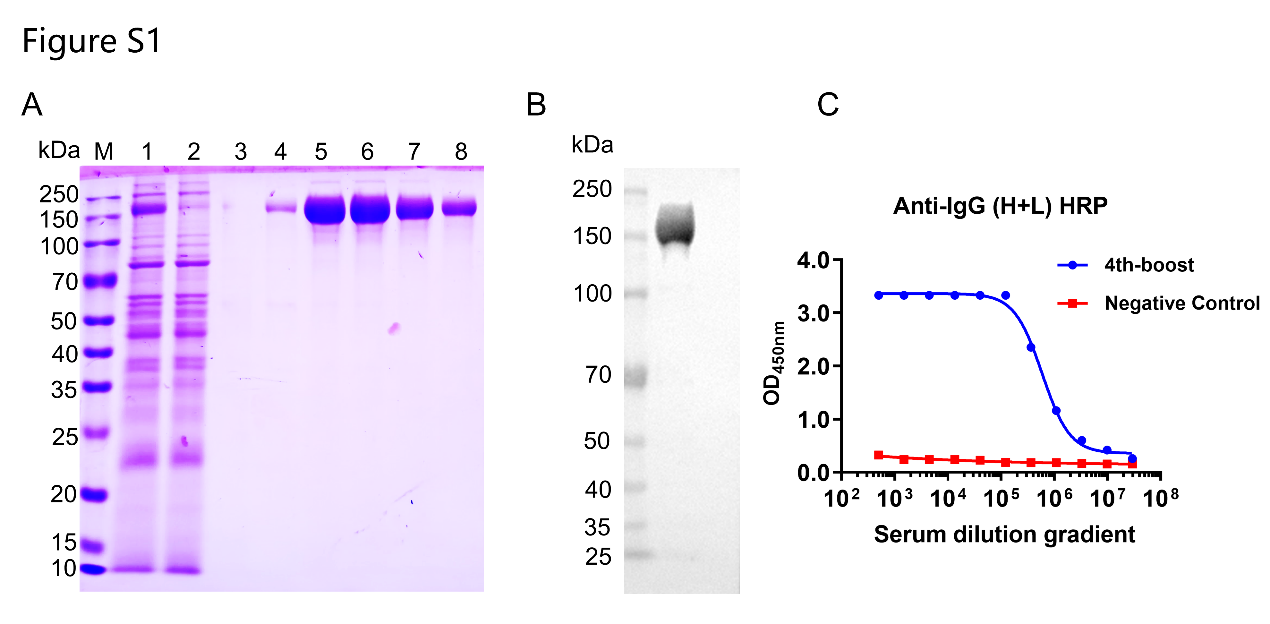


**Fig. S1:** Expression, purification, and identification of PDCoV-S protein. (A) SDS-PAGE migration profiles of purified recombinant proteins S (160 kDa). (B) Western blot analysis of the recombinant proteins S. Recombinant S protein were subjected to western blot analysis with mouse Anti-PDCoV-S mAb (1:5000) as primary antibody and HRP-conjugated goat anti-mouse as secondary antibody (1:10,000). (C) Titer of anti-PDCoV-S antibody in the immune paco serum detected by ELISA.


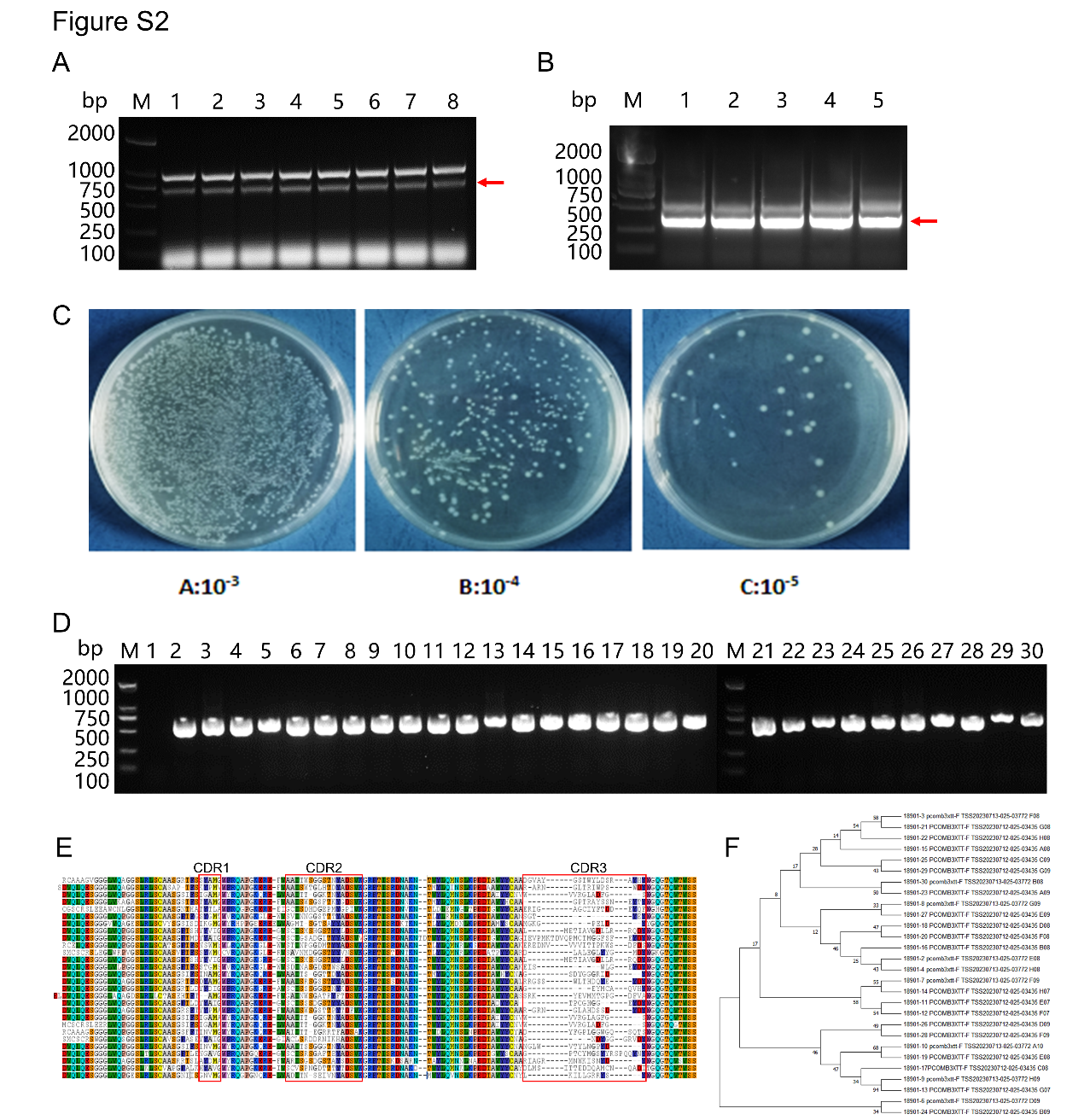


**Fig. S2:** Construction and identification of phage library. (A) Amplification of alpaca antibody fragment. (B) Amplification of VHH library fragments. (C) Determination of VHH library capacity. (D) The positive rate of VHH library was identified by PCR. (E) Amino acid sequence analysis of VHH antibody fragment containing correct reading frame. (F) Phylogenetic analysis of VHH amino acid sequences.


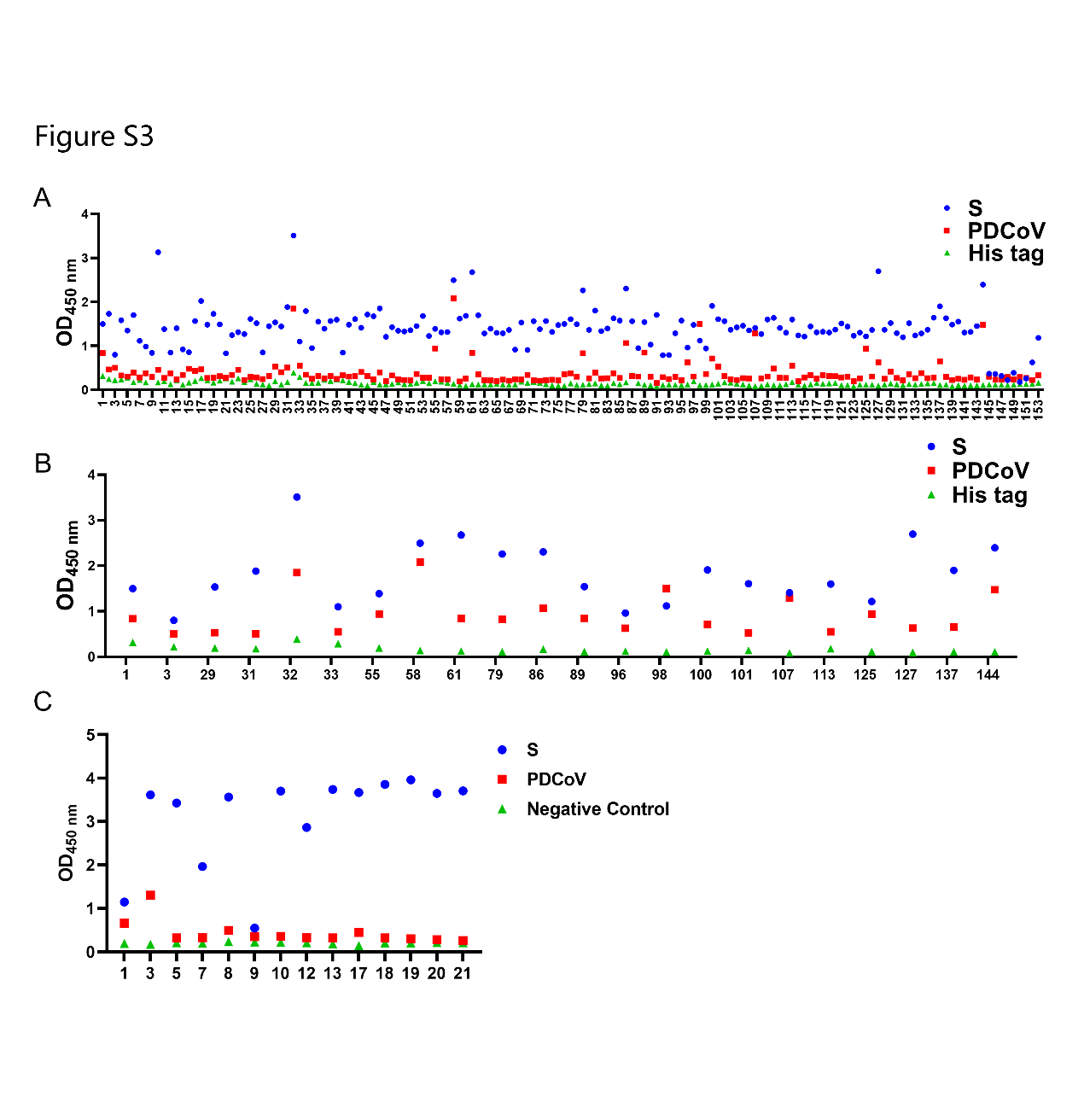


**Fig. S3:** Screening and identification of PDCoV-S protein-specific nanobody library. (A-B) Identification of PDCoV-S protein-binding phages by monoclonal phage ELISA. (C) Nanobodies specifically binding to PDCoV-S protein were screened by soluble ELISA.
